# Effectiveness of contemporary treatments for iatrogenic urethral strictures following endoscopic management of benign prostatic hyperplasia: a comprehensive review

**DOI:** 10.1007/s00345-026-06462-6

**Published:** 2026-05-21

**Authors:** Mattia Lo Re, Anna Cadenar, Marta Pezzoli, Elettra Fuligni, Behzad Abbasi, Łukasz Białek, Francesco Chierigo, Mikołaj Frankiewicz, Leonidas Karapanos, Jakob Klemm, Guglielmo Mantica, Paul Neuville, Bruno Bucca, Maciej Oszczudłowski, Elaine Redmond, Jordán Scherñuk, Juan Diego Tinajero, Wesley Verla, Andrea Minervini, Malte W. Vetterlein, Andrea Cocci

**Affiliations:** 1 Department of Experimental and Clinical Medicine, University of Florence, Florence, Italy; 2 Unit of Urology and Andrology, University of Florence, Careggi Hospital, 50100 Florence, Italy; 3 Department of Urology, University of California, San Francisco, CA USA; 4 Department of Urology, Centre for Postgraduate Medical Education, Warsaw, Poland; 5 Department of Urology, Department of Health Science, ASST Santi Paolo e Carlo, University of Milan, Milan, Italy; 6 Department of Urology, Medical University of Gdańsk, Gdańsk, Poland; 7 Department of Urology, Klinikum Leverkusen, Leverkusen, Germany; 8 Department of Urology, University Medical Center Hamburg-Eppendorf, Hamburg, Germany; 9 Department of Surgical and Diagnostic Integrated Sciences (DISC), University of Genoa, Genoa, Italy; 10 Department of Urology, Hospital Lyon Sud, Hospices Civils de Lyon, Lyon, France; 11 Department of Urology, Sapienza University of Rome, Policlinico Umberto I Hospital, Rome, Italy; 12 Department of Urology, Cork University Hospital, Cork, Ireland; 13 School of Medicine, University College Cork, Cork, Ireland; 14 Department of Urology, Hospital Italiano de Buenos Aires, Ciudad Autónoma de Buenos Aires, Argentina; 15 Chelsea Centre for Gender Surgery, Chelsea and Westminster Hospital, NHS Trust, London, UK; 16 Department of Urology, AZ Maria Middelares, Ghent, Belgium

**Keywords:** Urethral stricture, BPH, Transurethral surgery, Urethroplasty

## Abstract

**Purpose:**

Iatrogenic urethral strictures represent a relevant complication following endoscopic surgical treatment for benign prostatic hyperplasia, potentially leading to significant morbidity and impaired quality of life. Despite advances in endoscopic technologies, urethral trauma related to prolonged operative time and large-caliber instruments remains a concern. The optimal management of urethral strictures secondary to surgery is still debated, and high-quality evidence comparing endoscopic and open reconstructive approaches is lacking. This systematic review aimed to evaluate the efficacy and safety of contemporary treatments for iatrogenic urethral strictures following endoscopic management of benign prostatic hyperplasia.

**Materials and methods:**

A systematic review was conducted according to PRISMA guidelines and registered in PROSPERO (CRD42024604611). MEDLINE, Embase, and the Cochrane Library were searched from 2000 to June 2025. Studies reporting outcomes of endoscopic or open surgical treatments for urethral strictures following benign prostatic hyperplasia surgery were included. Data extraction focused on patient characteristics, stricture features, treatment modality, success rates, complications, and follow-up. Due to substantial heterogeneity, a meta-analysis was not performed.

**Results:**

Eleven studies comprising a total of 610 patients were included. Of these, 443 (73%) patients underwent primary treatment for urethral stricture, while 167 (27%) were treated for recurrent disease. The bulbar and membranous urethra were the most commonly involved sites. Overall, 58 patients (9%) underwent endoscopic management, whereas 552 patients (91%) were treated with open reconstructive surgery. The overall recurrence rate was 8 and 10% for both endoscopic and open approaches. However, follow-up duration differed substantially between groups, ranging from 12 to 24 months in endoscopic series and extending up to 54 months in open reconstructive cohorts. Complication rates were generally low. No perioperative or functional complications were reported following endoscopic treatments. Among open procedures, urinary incontinence was the most frequent complication, with rates varying from 4% after ventral onlay graft urethroplasty to 15% following intrasphincteric bulbo-prostatic anastomosis. Flap-related complications were rare.

**Conclusion:**

Management of urethral strictures following endoscopic prostatic surgery remains challenging. While endoscopic treatments offer acceptable short-term outcomes with minimal morbidity, open reconstructive surgery provides more durable long-term results at the cost of a higher, yet acceptable, risk of functional complications. Treatment choice should be individualized based on stricture characteristics, patient factors, and surgical expertise. Prospective, multicenter studies with standardized outcome reporting and longer follow-up are warranted to optimize treatment algorithms for this complex condition.

**Supplementary Information:**

The online version contains supplementary material available at 10.1007/s00345-026-06462-6.

## Introduction

Benign prostatic hyperplasia (BPH) is a common condition in older men, causing impairment to quality of life and overall health. Over the past decades, the incidence of Lower Urinary Tract Symptoms (LUTS) related to BPH has steadily increased: the age-standardised prevalence of BPH was reported up to 2480 (1940 to 3090) per 100,000 people [1]. When conservative pharmacological treatment is insufficient to relieve LUTS, surgical intervention for BPH is recommended, with transurethral resection of the prostate (TURP) and enucleation techniques being standard options, depending on gland size [2]. Despite recent advancements in surgical techniques and instruments for the management of BPH, these innovations have not necessarily translated into a reduced risk of complications such as iatrogenic urethral stricture formation. Endoscopic procedures are often characterized by prolonged operative times and the continued use of relatively large-caliber endoscopes (22–24 Fr), factors that may increase urethral manipulation and ischemic injury [3–5]. As a result, the potential impact of technological progress on minimizing urethral stricture-related complications may have been underestimated and remains insufficiently addressed: transurethral surgery remains the most common cause of iatrogenic urethral strictures, occurring in between 0.3% and 13% of patients [6–8]. 

Several risk factors have been identified, including operative time, postoperative infections, and the caliber of the endoscopic sheath, while no clear evidence has been found regarding the energy modality used [9–13].

Managing iatrogenic urethral strictures can be challenging and varies considerably depending on the characteristics of the stenosis, ranging from observation to endoscopic treatments and open urethroplasty procedures [14, 15].

To the best of our knowledge, there is no high-quality evidence assessing the effectiveness of the diverse treatment alternatives. Therefore, this systematic review aimed to evaluate the efficacy of the various treatment options for iatrogenic urethral strictures, secondary to endoscopic BPH surgery.

## Materials and methods

The study protocol is registered in the International Prospective Register of Systematic Reviews database (PROSPERO: CRD42024604611). This systematic review conforms to the guidelines of the Preferred Reporting Items for Systematic Reviews and Meta-Analyses (PRISMA) statement (Supplementary Table 1) [16, 17].

The primary outcome of our review was to evaluate the overall efficacy of both endourological and open treatment for iatrogenic urethral strictures after endoscopic BPH surgery. As a secondary analysis, we sought to evaluate the complication rate.

### Study selection

MEDLINE via PubMed, Embase and Cochrane library were searched between the year 2000 and June 30, 2025, to identify relevant studies on management of iatrogenic urethral stricture. The search term included: “prostatic hyperplasia”, “resection”, “enucleation”, “vaporization” and “urethral stricture”. Details regarding the search strategy are presented in Supplementary Appendix 1. Moreover, we searched the reference lists of the selected papers to find additional studies of interest. Two independent investigators (R.C., E.F.) screened titles and abstracts to identify eligible studies. Any disagreement was by consensus between the two investigators and a third author (M.L.R.).

### Inclusion and exclusion criteria

**Fig. 1:**
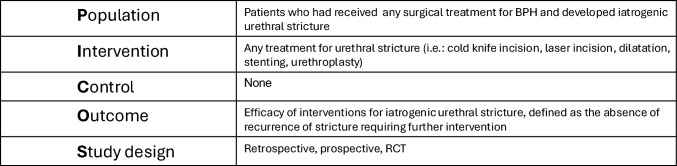
Population, interventions, comparator, outcomes, and study design (PICOS) framework to define the eligibility criteria; *RCTs* *randomized controlled trials*

We used the Population, Interventions, Comparator, Outcomes, and Study design (PICOS) framework to define the eligibility criteria (Fig. 1). Original articles describing treatment alternatives for urethral stricture secondary to prostatic surgery for BPH and assessing relative efficacy were included. We excluded non-English papers, reviews, letters, editorials, case reports, case series with less than 10 subjects, and conference papers.

### Data extraction

Two reviewers (R.C., E.F.) independently extracted data on study and patients’ characteristics, using Microsoft Excel software. Any disagreement was resolved by consensus between the two investigators and a third author (A.Ca.). From each study, we gathered essential details: first author’s name, publication year, country, number of centers involved, design of the study, number of patients, previous BPH surgery details, location of the urethral stricture, peri- and intra-operative variables regarding stricture treatment, success rate, and complications rate. Success rate was defined as freedom from stricture retreatment during study follow-up, accordingly with the last Delphi consensus about standardized outcomes after urethral stricture treatment [18].

### Quality assessment and risk of bias

Each study was evaluated by two investigators (A.Ca., M.L.R.) independently using the Cochrane Collaboration’s ROBINS-I tool for non-randomized studies [19]. Any discrepancy was resolved by consensus between the two investigators and a third author (A.Co.). Publication bias was not formally assessed because of the limited number and heterogeneity of the included studies.

### Data synthesis

Due to the heterogeneity of the available data, according to the EAU methodology [20], it was not possible to perform a meta-analysis. The extracted data were reported in the text and tables as shown in the original articles; however, when deemed necessary to obtain a better overview, relative frequencies were calculated using IBM SPSS Statistics version 29 (IBM Corp., Armonk, NY, USA).

## Results

### Study selection and characteristics

**Fig. 2:**
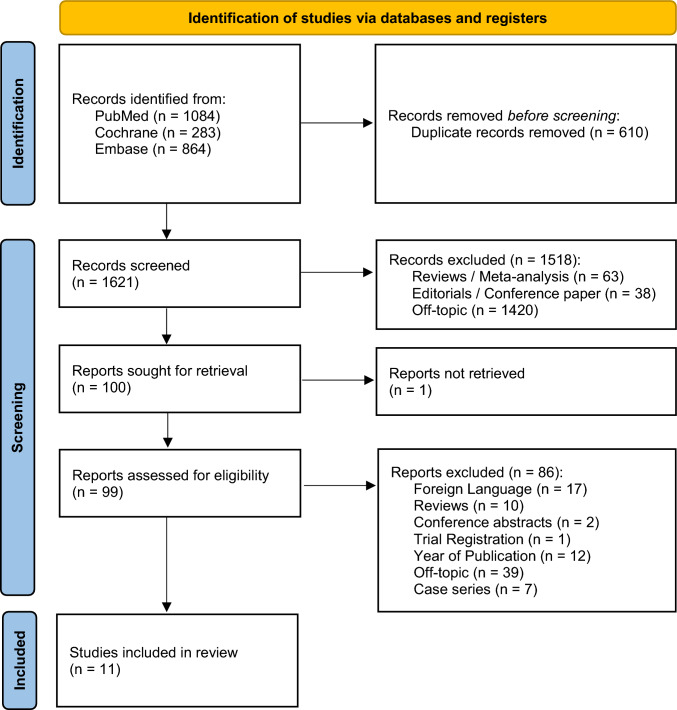
PRISMA selection flow-chart. Source: Page MJ, et al. BMJ 2021;372:n71. https://doi.org/10.1136/bmj.n71. This work is licensed under CC BY 4.0. To view a copy of this license, visit

Our initial search identified 2231 records. After duplicate removal, 1621 records were left for title and abstract screening. Based on the inclusion and exclusion criteria, 11 studies comprising 610 patients were eligible for analysis. Details regarding the selection process are presented in Fig. 2. Main characteristics of the included studies and clinical features are summarized in Tables 1 and 2, respectively [21–30].

**Table 1:** Characteristic of included studies

Authors	Study design	Country	Number of institution	Years of observation	Number of cases	Mean Age (SD)	Previous BPH surgery
Abu Nasra, 2020, [30]	Retrospective	Israel	1	2013—2014	14	76 (na)	TURP
Barbagli, 2019, [21]	Retrospective	Italy, India	2	2002—2017	69	61.5 (12.5)	63 TURP 3 HOLEP 3 TUIP
Borkowski, 2019, [22]	Retrospective	Poland	1	1994—2015	19	Not specified	19 OSP
Elsaqa, 2022, [23]	Retrospective	USA	1	2015—2021	20	72.25 (2.75)	HOLEP
Favre, 2021 [24]	Retrospective	Argentina	1	2011—2019	77	Not specified	34 TURP 26 HOLEP 17 OSP
Gomez, 2021 [25]	Prospective	Chile	1	2010—2019	40	65	18 TURP 22 OSP
Joshi, 2023 [26]	Prospective	India	1	2014—2020	17	62.25 (9.25)	16 TURP 1 HOLEP
Kore, 2023 [27]	Retrospective	India	1	2016—2019	76	69 (6)	27 mTURP 41 bTURP 8 HOLEP
Onol, 2008 [28]	Retrospective	Cyprus	1	1997—2007	26	59 (6.75)	21 TURP 5 OSP
Yagi, 2022 [29]	Retrospective	Japan	2	2011—2021	82	71.5 (5.93)	52 TURP 24 HOLEP 6 vaporitazion
Kulkarni, 2019 [31]	Prospective	India	1	2010–2017	170	67.3 (54–87)	TURP

*BPH* benign prostatic hyperplasia, *TURP* trans-urethral resection of the prostate, *HoLEP* holmium laser enucleation of the prostate, *TUIP* trans-urethral incision of the prostate, *OSP* open simple prostatectomy, *mTURP* monopolar TURP, *bTURP* bipolar TURP

**Table 2:** Strictures details and treatment of choice

Author	Number of cases	Follow-up months	Number of strictures	Stricture location	Stricture length, mean (mm)	Previous stricture treatment	Actual treatment for stricture
Abu Nasra, 2020 [30]	14	24	Single	Not specified	< 10	No	Cold knife urethrotomy
Barbagli, 2019 [21]	69	77.25 (± 43.9)	Single	Bulbar urethra Membranous urethra	Not specified	36 naïve 33 DVIU	Modified ventral onlay graft urethroplasty
Borkowski, 2019 [22]	19	Not specified	Multiple	Bulbar urethra Membranous urethra Prostatic urethra Bladder neck	Not specified	No	Cold knife urethrotomy
Elsaqa, 2022 [23]	20	20	Single	Prostatic urethra	Not specified	No	Dilation Cold knife urethrotomy
Favre, 2021 [24]	77	52.5 (± 14.29)	Single	Bulbar urethra Membranous urethra	15 (± 7.4)	30 naïve 14 dilation 20 DVIU 3 urethroplasty	Bulbo-membranous anastomosis
Gomez, 2021 [25]	40	53 (± 27.65)	Single	Bulbar urethra Membranous urethra Prostatic urethra	26	17 naïve 11 dilatation 12 DVIU	Intrasphincteric bulbo‑prostatic anastomosis
Joshi, 2023 [26]	17	36 (± 17.46)	Single	Bulbar urethra	40 (± 29.6)	5 naïve 2 dilation 10 DVIU	Double-face buccal mucosa graft
Kore, 2023 [27]	76	Not specified	Single	Fossa navicularis Bulbar urethra Membranous urethra	Not specified	No	Ventral onlay buccal mucosal graft
Onol, 2008 [28]	26	30.2 (± 22.55)	Single	Fossa navicularis	Range: 0.6–1.4	DIlation	Ventral transverse fasciocutaneous penile flap
Yagi, 2022 [29]	76	Not specified	Single	Fossa navicularis Bulbar urethra Membranous urethra Bladder neck	15 (± 11.11)	3 naïve 73 DVIU/dilatation 6 urethroplasty	6 Ventral meatotomy 1 Meatoplasty 1 Transurethral ventral inlay 11 onlay augmentation 13 staged urethroplasty 19 perineal urethrostomy 18 anastomosis 7 non-transecting urethroplasty
	6		Multiple				1 EPA + PU 1 2 NTU + PU 2 NTU + onlay augmentation 1 PU
Kulkarni, 2019 [31]	170	14 [6, 192]	Not specified	Bulbar urethra Penile urethra Penile + Bulbar urethra Panurethral	Bulbar: 1.95 cm Penile: 4.65 cm Penile + bulbar: 4.75 cm Panurethral: 16.75 cm	Naïve	Dorsal onlay BMG urethroplasty 94 Ventral onlay BMG urethroplasty 71 Distal dorsal + proximal ventral BMG 5 Endoscopic management 5

*DVIU* direct visual internal urethrotomy, *TUR-BN* trans-urethral resection of bladder neck, *TUI-BN* trans-urethral incision of bladder neck, *EPA* excision and primary anastomosis urethroplasty, *PU* perineal urethrostomy*, NTU* non transecting urethroplasty*, BMG* buccal mucosal graft

Of the 11 studies included, 3 were prospective studies and 8 were retrospective series.

### Risk of bias assessment

Supplementary Fig. 2 illustrates authors’ assessment of each domain for the included studies.

### Stricture characteristics and failure rate

A total of 610 patients were evaluated. Of those, 443 (73%) underwent a primary surgical procedure for urethral stricture after BPH treatment, whereas 167 (27%) were facing a relapse from a previous treatment.

Regarding the localization, the bulbar urethra was the most affected, reported as involved in 7 studies [21, 22, 24–27, 29, 31], the membranous urethra was involved in 6 studies [21, 22, 24, 25, 27, 29], while the prostatic urethra was affected in 3 studies [22, 23, 25]. The fossa navicularis was involved in 3 studies [27–29], Kulkarni et al. reported cases of penile and paurethral strictures [31], while Abu Nasra et al. did not specify the localization of the stricture [30].

A total of 67 (11%) recurrences were reported across 10 studies (Table 3), with a rate of failure spreading from 4% up to 18%. In only one study wasn’t reported any relapse during the 24-month follow-up period [30].

**Table 3:** Failure rate and re-treatment choice

Author	Number of cases	Follow-up months	Number of recurrence, (%)	Months to recurrence (mean)	Treatment for second recurrence
Barbagli, 2019 [21]	69	77.25 (± 43.9)	11 (17%)	Not specified	3 DVIU 2 dilatation 6 associated 3 none
Borkowski, 2019 [22]	19	Not specified	1 (5%)	Not specified	DVIU
Elsaqa, 2022 [23]	20	20	3 (15%)	11	DVIU
Favre, 2021 [24]	77	52.5 (± 14.29)	3 (4%)	6 (± 2)	1 DVIU 1 BMA 1 Urethroplasty
Gomez, 2021[25]	40	53 (± 27.65)	4 (10%)	12 (± 2.94)	3 Dilatation 1 DVIU
Joshi, 2023 [26]	17	36 (± 17.46)	2 (12%)	< 12	1 Dilatation 1 DVIU
Kore, 2023 [27]	76	Not specified	8 (10%)	Range 6–15	DVIU
Onol, 2008 [28]	26	30.2 (± 22.55)	1 (4%)	3	Fistula repair
Yagi, 2022 [29]	82	Not specified	4 (5%)	24.75 (± 32.1)	DVIU
Kulkarni, 2019 [31]	170	14 [6, 192]	30 (18%)	Not specified	Not specified

*DVIU* direct visual internal urethrotom*, BMA* buccal mucosal graft augmentation

Follow-up duration was heterogeneously reported across the included studies. Among endoscopic series, follow-up ranged from a fixed 24 months in Abu Nasra et al. [30] to a minimum follow-up of at least 12 months in Elsaqa et al. [23], while Borkowski et al. [22] did not report a specific follow-up duration. In contrast, studies evaluating open reconstructive surgery generally provided longer follow-up, reported either as median values—52 months in Barbagli et al. [21], 53 months in Favre et al. [24], 24 months in Joshi et al. [26], and 43 months in Yagi et al. [29]—or as mean follow-up, including 14 months in Kulkarni et al.[31], 53 months in Gómez et al. [25], 34 months in Kore [27], and 30.2 months in Onol et al. [28].

### Surgical approach

Overall, 4 and 8 authors described endoscopic [22, 23, 30, 31] and open procedures [21, 24–29, 31], for a total of 58 and 552 patients, respectively.

With regard to endoscopic surgery, 58 (100%) subjects underwent cold knife urethrotomy with or without dilatation. Among these procedures, a total of 4 (7%) failures were recorded [22, 23].

Of the 552 open procedures, 117 (21%) patients in two papers underwent Excision and Primary Anastomosis (EPA) urethroplasty and the remaining 435 (79%) underwent urethroplasty with mucosal graft. A total of 63 (10%) recurrences were reported [21, 24–29, 31]. 

### First and re-do treatments

Five studies analyzed only naïve patients for urethral stricture treatment. While Abu Nasra et al. [30], Borkowski et al. [22], and Elsaqa et al. [23] treated these patients with cold knife urethrotomy, Kore et al. [27] treated patients with urethroplasty using ventral onlay buccal mucosal graft (Table 2). Kulkarni et al. [31] reported a mixed strategy, in which only 5 patients with complete obliterate stricture at the membranous urethra were treated with cold knife urethrotomy and then maintained a regimen of self-intermittent catheterization; other cases were treated with urethroplasty.

Treatment failure reported was 0 (0%), 1 (5%), 3 (15%), 8 (10%), 30 (18%), respectively (Table 3).

All authors that specified this data, treated relapses with DVIU.

One study included only non-naïve patients. Onol et al. [28] analyzed 26 cases of fossa navicularis reconstruction with a ventral transverse island fasciocutaneous penile flap, recording only 1 (3.8%) relapse after 3 months from the procedure. All patients were previously treated with meatal dilatation.

The remaining five studies included both first and subsequent treatments for urethral stricture, for a total of 118 and 167 patients, respectively [21, 24–26, 29]. Most authors did not evaluate the impact of previous relapses on treatment failure rate.

Barbagli et al. [21] did not find any differences in success rate according to previous urethrotomies. The authors described a modified ventral onlay graft urethroplasty in 69 patients, of which 36 (52%) were first procedures and 33 (48%) re-do treatments. Eleven (16%) failures were reported. 3 patients suffered from post-procedural urinary incontinence.

### Complications

Across the included studies, complication rates varied substantially according to the type of procedure performed (Table 4). Endoscopic treatments were generally associated with low reported morbidity, with no perioperative or functional complications reported in the available series. Among reconstructive procedures, Barbagli et al. showed a low incidence of urinary incontinence (4%) [21], whereas higher rates were observed after bulbo-membranous anastomosis (8%) [24] and intrasphincteric bulbo-prostatic anastomosis (15%) [25]. Flap-related complications were uncommon, with penile fasciocutaneous flap necrosis reported in 4% of cases in the series of Onol et al. [28]. In most series evaluating open surgical techniques, including buccal mucosa graft urethroplasty, perineal urethrostomy, and anastomotic repairs, no perioperative or functional complications were reported [22, 23, 26, 27, 29–31].

**Table 4:** Complications rate

Author	Number of cases	Follow-up months	Actual treatment for stricture	Complication reported	N, Rate (%)
Abu Nasra, 2020 [30]	14	24	Cold knife urethrotomy	No complication	0
Barbagli, 2019 [21]	69	77.25 (± 43.9)	Modified ventral onlay graft urethroplasty	Urinary incontinence	3 (4%)
Borkowski, 2019 [22]	19	Not specified	Cold knife urethrotomy	No complication	0
Elsaqa, 2022 [23]	20	20	Dilation Cold knife urethrotomy	No complication	0
Favre, 2021 [24]	67	52.5 (± 14.29)	Bulbo-membranous anastomosis	Urinary incontinence	6 (8%)
Gomez, 2021 [25]	40	53 (± 27.65)	Intrasphincteric bulbo‑prostatic anastomosis	Urinary incontinence	6 (15%)
Joshi, 2023 [26]	17	36 (± 17.46)	Double-face buccal mucosa graft	No complication	0
Kore, 2023 [27]	76	Not specified	Ventral onlay buccal mucosal graft	No complication	0
Onol, 2008 [28]	26	30.2 (± 22.55)	Ventral transverse fasciocutaneous penile flap	Flap necrosis	4%
Kulkarni, 2019 [31]	170	14 [6, 192]	Dorsal, ventral and mixed onlay BMG urethroplasty Endoscopic management	No complications	0
Yagi, 2022 [29]	76	Not specified	6 Ventral meatotomy 1 Meatoplasty 1 Transurethral ventral inlay 11 onlay augmentation 13 staged urethroplasty 19 perineal urethrostomy 18 anastomosis 7 non-transecting urethroplasty	No complication	0
	6		1 EPA + PU 1 2 NTU + PU 2 NTU + onlay augmentation 1 PU		

*DVIU* direct visual internal urethrotomy, *TUR-BN* trans-urethral resection of bladder neck, *TUI-BN* trans-urethral incision of bladder neck, *EPA* excision and primary anastomosis urethroplasty, *PU* perineal urethrostomy, *NTU* non-transsecting urethroplasty

## Discussion

This systematic review summarizes the current evidence on treatments for iatrogenic stenoses and strictures of the lower urinary tract following surgical management of BPH based on 11 studies with a total of 610 patients. Several key observations can be made.

Firstly, the overall recurrence rate after intervention was 8–10% for endoscopic treatment and open surgery, though results varied according to treatment type and stricture location.

Despite an overall comparable failure rate between studies reporting endoscopic treatments and those reporting open urethroplasty, follow-up duration differs significantly. Minimally invasive endoscopic approaches achieved moderate short-term success, with a follow up spreading from 12 to 24 months, whereas open urethroplasty provided far more durable outcomes at double time follow-up, from 24 up to 54 months. This is consistent with prior data indicating substantially higher long-term patency rates for urethroplasty (on the order of 82.4–83.5%) compared to internal urethrotomy (32.2%) [32]. Moreover, no clear advantage emerged for laser over cold-knife urethrotomy, confirming that the application of an energy source does not significantly affect stricture recurrence [33, 34].

Secondly, a history of previous interventions does not seem to negatively impact outcomes. Barbagli et al*.* reported similar success rates between primary urethroplasty and repeat urethroplasty in recurrent cases [21]. Notably, Barbagli’s series showed that a well-performed urethroplasty can achieve high cure rates regardless of previous endoscopic failures; therefore, in skilled hands, urethroplasty may even be considered a first-line treatment option for suitable strictures. Additionally, some evidence indicates that the cause of the stricture can affect recurrence: strictures after HoLEP tend to have lower recurrence rates than those after TURP, possibly due to less extensive thermal injury from enucleation [23]. A recent meta-analysis by Pirola et al*.* [35] confirmed that the incidence of urethral stricture following TURP is higher than after HoLEP. It's plausible that the increased thermal damage from TURP causes more significant scarring, potentially hindering healing after stricture repair; however, further research is needed to verify this.

Thirdly, among open reconstructive procedures, urinary incontinence emerged as the most relevant functional complication, although its incidence varied substantially according to the surgical technique employed. The relatively low incontinence rate reported by Barbagli et al. (4%) following ventral onlay graft urethroplasty and higher incontinence rates observed after bulbo-membranous anastomosis (8%) and intrasphincteric bulbo-prostatic anastomosis (15%) highlight the intrinsic risk associated with reconstruction in close proximity to the external sphincter, particularly in patients with prior BPH surgery and compromised internal sphincter function. Datas are consistent with the literature, as reported that incontinence occurs in up to 14% with bladder neck reconstruction and up to 25% after reconstruction of BMS after previous surgery for BPH [15, 36].

This systematic review has some limitations. First, the evidence is limited by retrospective study designs, small sample sizes, and diverse patient characteristics. Follow-up periods were often short (12–24 months), which might underestimate late recurrences, and success criteria varied widely. Second, functional outcomes, including continence and sexual function, were reported inconsistently, although incontinence after urethroplasty was noted in about 4 to 17% of cases when documented.

Future studies should adopt prospective, multicenter study designs with standardized outcome definitions and extended follow-up periods to more accurately determine long-term failure-free survival and functional results.

Overall, our findings confirmed that definitive open surgery (urethroplasty or bladder neck reconstruction) leads to slightly superior long-term outcomes for post-BPH urethral stricture. Endoscopic procedures are still a choice, but surgeons and patients should be aware of a higher recurrence rate. The choice of technique should be individualized based on stricture characteristics, patient comorbidities, and the expertise available, considering that urethroplasty could lead to longer failure-free follow-up.

## Conclusion

This systematic review highlights that iatrogenic urethral strictures following surgical treatment for benign prostatic hyperplasia remain a clinically relevant and challenging condition. Endoscopic management, including internal urethrotomy and dilation, is associated with acceptable short-term outcomes but carries a slightly higher risk of recurrence compared with open reconstructive surgery. Urethroplasty consistently achieves long-term patency, with success rates exceeding 80–90% in most contemporary series.

Future prospective studies with direct comparison, standardized outcome measures and longer follow-up are needed to refine treatment algorithms and optimize long-term functional outcomes and long-term patency.

## Supplementary Information

Below is the link to the electronic supplementary material.

Supplementary file1 (DOCX 15 KB)

Supplementary file2 (DOCX 29 KB)

## Data Availability

No datasets were generated or analysed during the current study.
