# Supplementary material for: Effectiveness of contemporary treatments for iatrogenic urethral strictures following endoscopic management of benign prostatic hyperplasia: a comprehensive review
Source: World J Urol. 2026 May 21;44(1):373. doi: 10.1007/s00345-026-06462-6 (PMC13194237; doi:10.1007/s00345-026-06462-6)
Supplement: Supplementary file 1 — Supplementary file1 (DOCX 15 KB) [file 345_2026_6462_MOESM1_ESM.docx]

| #1 | ("Prostatic Hyperplasia"[Mesh] OR BPH[TIAB] OR (prostat*[tiab] AND (adenoma*[TIAB] OR hyperplas*[TIAB]  OR enlarg*[TIAB]  OR hyperthrop*[TIAB]  OR obstruct*[TIAB])) OR benign prostatic hyperplasia OR BPE[TIAB]) |  |
| --- | --- | --- |
| #2 | (Prostate resection OR (Prostat*[tiab] AND (enucleat*[tiab] OR vaporiz*[tiab] OR resect*[tiab])) OR "monopolar transurethral"[tiab] OR "bipolar transurethral"[tiab] OR "MTURP"[tiab] OR "BTURP"[tiab] OR "plasmakinetic"[tiab] OR "PKRP"[tiab] OR "PKEP"[tiab] OR "PKERP"[tiab] OR "TURis"[tiab] OR “Transurethral resection in saline”[tiab] OR photovaporiz* OR “photo vaporiz*”  OR "PVP"[tiab] OR "PVEP"[tiab] OR "holmium"[tiab] OR "HoLEP"[tiab] OR "thulium"[tiab] OR "ThuLEP"[tiab] OR "ThuVEP"[tiab] OR "diode"[tiab] OR "DiLEP"[tiab] OR "BTUEP"[tiab] OR "MTUERP"[tiab] OR "RASP"[tiab] OR “Robot assisted Simple Prostatectomy”[tiab]) |  |
| #3 | (Urethral stenosis OR (urethr*[tiab] AND (scleros*[tiab] OR obstruct*[tiab] OR stenos*[tiab] OR contract*[tiab] OR stricture*[tiab] OR narrow*[tiab]))) |  |
| #4 | #1 AND #2 AND #3 |  |
|  |  |  |
