# Supplementary material for: Effectiveness of contemporary treatments for iatrogenic urethral strictures following endoscopic management of benign prostatic hyperplasia: a comprehensive review
Source: World J Urol. 2026 May 21;44(1):373. doi: 10.1007/s00345-026-06462-6 (PMC13194237; doi:10.1007/s00345-026-06462-6)
Supplement: Supplementary file 2 — Supplementary file2 (DOCX 29 KB) [file 345_2026_6462_MOESM2_ESM.docx]

Risk Of Bias In Non-randomised Studies - of Interventions

| **Author** | **D1** | **D2** | **D3** | **D4** | **D5** | **D6** | **D7** |
| --- | --- | --- | --- | --- | --- | --- | --- |
| Abu Nasra, 2020 | moderate | low | moderate | low | moderate | serious | moderate |
| Barbagli, 2019 | serious | moderate | moderate | serious | low | serious | moderate |
| Borkowski, 2019 | moderate | moderate | moderate | low | serious | serious | moderate |
| Elsaqa, 2022 | moderate | moderate | serious | low | moderate | low | low |
| Favre, 2020 | moderate | moderate | moderate | low | moderate | moderate | moderate |
| Gomez, 2021 | serious | moderate | low | low | low | moderate | moderate |
| Joshi, 2021 | moderate | moderate | moderate | low | low | moderate | low |
| Kore, 2023 | serious | serious | low | serious | low | moderate | moderate |
| Onol, 2008 | serious | moderate | moderate | low | low | moderate | moderate |
| Yagi, 2022 | moderate | low | moderate | moderate | low | serious | serious |
| Kulkarni, 2019 | Moderate | Low | Moderate | Moderate | Low | Serious | low |
